# 2177. Institutional Prevalence of Drug-Resistant Pathogens in Community-Acquired Pneumonia

**DOI:** 10.1093/ofid/ofac492.1797

**Published:** 2022-12-15

**Authors:** Helen Ding, Jordan Loomis, Jessica Ortwine, Norman S Mang, Wenjing Wei, Bonnie C Prokesch, Nainesh Shah, Ellen O'Connell

**Affiliations:** Parkland Health, Dallas, Texas; Parkland Health, Dallas, Texas; Parkland Health, Dallas, Texas; Parkland Health, Dallas, Texas; Parkland Health, Dallas, Texas; Parkland Health, Dallas, Texas; UT Southwestern, Dallas, Texas; UT Southwestern, Dallas, Texas

## Abstract

**Background:**

Community-acquired pneumonia (CAP) is a leading cause of hospitalizations and plays a major role in mortality. The previous Infectious Diseases Society of America (IDSA) definition of healthcare-associated pneumonia (HCAP) was found to be neither sensitive nor specific for identifying drug-resistant pathogens including methicillin-resistant *Staphylococcus aureus* (MRSA) and *Pseudomonas aeruginosa* (PsA) as a cause of CAP, and use is no longer supported by current guidelines. The 2019 IDSA guidelines for CAP management emphasizes the need for clinician understanding of local epidemiology data to guide selection of appropriate treatment. The primary objective of this study was to determine the prevalence of MRSA and PsA CAP at our institution.

**Methods:**

This was a retrospective observational study of patients admitted to our 870-bed public hospital with a CAP or HCAP diagnosis within 48 hours of admission between March 2016 and March 2021. The primary outcome of prevalence of CAP caused by MRSA or PsA was determined by comparing the number of blood and adequate sputum cultures with MRSA or PsA to total reviewed cases. Secondary outcomes included the percentage of initial antibiotic regimens involving a broad-spectrum agent, percentage of initial broad-spectrum regimens de-escalated within 72 hours if indicated, and duration of CAP antibiotic treatment.

**Results:**

A total of 220 patients were included. MRSA or PsA was isolated in 1.36% of adequate sputum cultures collected (n=3/35) and in no collected blood cultures (n=0/208). The local prevalence of CAP caused by MRSA or PsA among the analyzed sample was 1.36% (n=3/220) (**Table 1**). MRSA nares screening tests were completed in 10% of cases, 4.5% of which were positive (n=1/22). Secondary end point results are presented in **Table 2.**

**Table 1.**
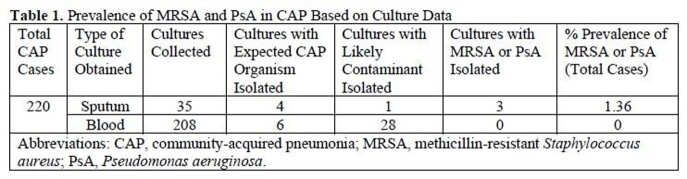
Prevalence of MRSA and PsA in CAP Based on Culture Data

**Table 2.**
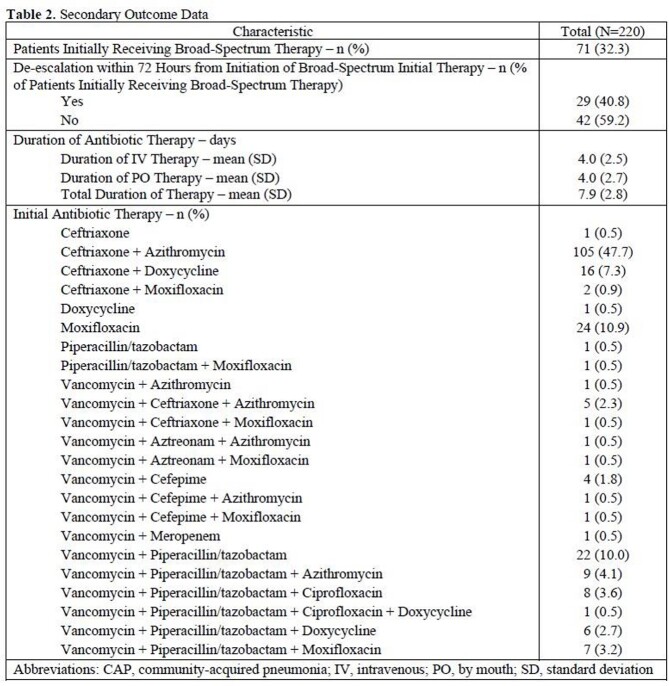
Secondary Outcome Data

**Conclusion:**

The overall prevalence of CAP caused by MRSA or PsA among admitted patients is low at Parkland Hospital. Further research is needed to identify local risk factors associated with CAP caused by drug-resistant pathogens.

**Disclosures:**

**All Authors**: No reported disclosures.

